# Intentional avoidance of the esophagus using intensity modulated radiation therapy to reduce dysphagia after palliative thoracic radiation

**DOI:** 10.1186/s13014-017-0771-6

**Published:** 2017-01-26

**Authors:** Patrick V. Granton, David A. Palma, Alexander V. Louie

**Affiliations:** 1 Department of Radiation Oncology, London Health Sciences Centre, Commissioners Road East, N6A 4 L6 London, ON Canada; 2 Department of Epidemiology and Biostatics, Western University, London, ON Canada

**Keywords:** Dysphagia, Esophagitis, Normal tissue complication probability, Lyman-Kutcher-Burman model, Palliative care

## Abstract

**Background:**

Palliative thoracic radiotherapy is an effective technique to alleviate symptoms of disease burden in advanced-stage lung cancer patients. Previous randomized controlled studies demonstrated a survival benefit in patients with good performance status at radiation doses of 35Gy_10_ or greater but with an increased incidence of esophagitis. The objective of this planning study was to assess the potential impact of esophageal-sparing IMRT (ES-IMRT) compared to the current standard of care using parallel-opposed pair beams (POP).

**Methods:**

In this study, 15 patients with lung cancer treated to a dose of 30Gy in 10 fractions between August 2015 and January 2016 were identified. Radiation treatment plans were optimized using ES-IMRT by limiting the max esophagus point dose to 24Gy. Using published Lyman-Kutcher-Burman normal tissue complication probabilities (LKB-NTCP) models, both plans were evaluated for the likelihood of esophagitis (≥ grade 2) and pneumonitis (≥ grade 2).

**Results:**

Using ES-IMRT, the median esophageal and lung mean doses reduced from 16 and 8Gy to 7 and 7Gy, respectively. Using the LKB models, the theoretical probability of symptomatic esophagitis and pneumonitis reduced from 13 to 2%, and from 5 to 3%, respectively. The median normalize total dose (NTD mean) accounting for fraction size for the GTV and PTV of the clinically approved POP plans compared to the ES-IMRT plans were similar.

**Conclusion:**

Advanced radiotherapy techniques such as ES-IMRT may have clinical utility in reducing treatment-related toxicity in advanced lung cancer patients. Our data suggests that the rate of esophagitis can be reduced without compromising local control.

## Background

Palliative radiotherapy can be an effective modality to alleviate and prevent symptoms related to advanced stage non-small cell lung cancer (NSCLC). In this setting, a meta-analysis of randomized controlled trials (RCTs) concluded that higher palliative radiation doses (specifically, exceeding a dose of 35 Gy_10_) were associated with a modest improvement in overall survival, but at the cost of increased rates of dysphagia [1]. These RCTs were conducted in an era where radiation delivery for these patients was limited to simple beam arrangements, e.g. a parallel-opposed pair (POP).

Since then, there have been a number of advances in radiation planning and delivery, such as intensity modulated radiotherapy (IMRT), which has been widely adopted and implemented in the curative setting for a number of treatment sites where normal tissue toxicity may present a limiting factor such as in head and neck cancers, for example [2]. IMRT permits a more conformal radiation dose through treatment planning optimization and field modulation but is needs greater resources, time, and costs.

While IMRT is not routinely used in the palliative lung radiotherapy setting, it could be employed to minimize dose to the esophagus, which would typically receive the full treatment dose in a POP beam arrangement delivered in the common scenario of centrally located lung cancer and/or bulky mediastinal lymphadenopathy causing symptoms. A recent study described and reported the clinical IMRT treatment approach in 20 curative-intent NSCLC patients whereby the contralateral esophagus wall was spared without compromising planning target volume (PTV) coverage with favourable results [3].

Under the auspices of the Canadian Pulmonary Radiotherapy Group (CAPRI, http://www.capriclinicaltrials.com/), patients with advanced-stage NSCLC patients are being recruited to a randomized phase III trial, PROACTIVE (NCT02752126), comparing palliative POP radiotherapy versus esophageal-sparing IMRT (ES-IMRT). As the use of ES-IMRT in PROACTIVE has implications of underdosing of PTV and increased dose to other organs at risk (OARs), the purpose of this planning study is to assess the feasibility of esophageal-sparing IMRT (ES-IMRT), and the potential benefits of ES-IMRT compared to the current standard of treatment.

## Methods

In this institutional research ethics board-approved study (Western University, REB ID: 107547) patients with advanced lung cancer treated with standard palliative radiotherapy (30 Gy in 10 fractions) between August 2015 and January 2016 were identified. Patients were eligible for study inclusion if the standard-of-care POP patient plans contained at least 5 cm of the esophagus within the treatment field. The standard approach for palliative planning employs a fast helical scan with a 5 mm CTV isotropic expansion for microscopic disease and a 5 mm PTV margin. As the final decision of treatment intent was unclear at the time of their CT simulation scan a small number of patients had a 4DCT performed. In this situation the ITV was expanded by 10 mm or more (chosen expansion at the discretion of the treating oncologist) to form the PTV. In either situation, the GTV or ITV was examined or modified to ensure no overlap with the defined (normal) esophagus contour. The field borders in either simulation situation were at least 5 mm away from the PTV according to the digitally reproduced radiograph. Multi-leaf collimators were used to shield organs-at-risk (OAR), which included, the heart, healthy lung (defined as left and right lung minus GTV), and cord.

A separate optimized esophageal-sparing (ES-IMRT) treatment was created using a Volumetric Modulated Arc Therapy (VMAT) approach with the goal of limiting the max esophagus point dose to 80% of the prescription (i.e. 24 Gy). To encourage a dose falloff for tumors that were near or abutting the esophagus, a 5-mm ring was created around the esophagus as an optimization structure. For portions of the PTV that overlapped with the esophagus and esophagus ring, the PTV coverage was compromised to allow a minimum of 20 Gy and the remaining planning target volume the ESPTV was used as a standard optimizing structure.

The dose to 95% of the volume (D95) contained within ESPTV was required to be equal or better than the D95 of the PTV in the clinically delivered POP. If any portion of the GTV was within this ring, the coverage was compromised such that no more than 1 cubic centimetre dose could receive lower than 80% of the intended prescription. To prevent dose from spilling into the healthy lung (defined as left and right lung minus the GTV), the volumes receiving 5 and 16 Gy (i.e.V5, and V16) of the lung were required to be less than 60 and 35%, respectively. No contiguous 2 cc volume was allowed to exceed 115% of the prescribed 30 Gy in either plan. Treatment was calculated over one or two arcs with a collimator rotations ranging between 10 and 45° and 315 to 350° using Pinnacle 9.10 (Philips, Eindhoven, The Netherlands).

Competing treatment plans were evaluated using published Lyman-Kutcher-Burman normal tissue complication probabilities (LKB-NTCP) models [4]. As there are a number of publically available LBK-NTCP models, we choose the two models that closely represented our treatment (e.g. without concurrent chemotherapy), patient cohort, and primary endpoints of esophagitis (≥ grade 2, RTOG) [5] and pneumonitis (≥ grade 2, RTOG) [6]. The input parameters for the LKB models were TD50 = 44.9, *n* = 0.34, *m* = 0.34 and TD50 = 29.9, *n* = 1, *m* = 0.41 for esophagitis and pneumonitis, respectively. Earlier LKB-models with different parameters for esophagitis by Belderbos et al. [7] and Chapet et al. [8] were evaluated for completeness. The NTCP model was calculated as outlined by Mohan et al. accounting for differences in dose per fraction according to the following equations [4]:

$$ \begin{array}{l} NTCP = \frac{1}{\sqrt{2\pi}}\cdot {\displaystyle \underset{-\infty }{\overset{t}{\int }}}\left({e}^{\frac{x^2}{2}}\right) dx\\ {}\kern2.04em  t = \frac{D_{e ff}- T{D}_{50}}{mT{D}_{50}}\\ {}\kern0.84em {D}_{e ff} = {\left({\displaystyle \sum_i}{v}_i{D}_i^{1/ n}\right)}^n\end{array} $$

whereby m and n are the measure of the sigmoid slope and volume effect, respectively. TD50 is the uniform dose to an organ that results in a 50% complication rate and v_*i*_ is the fractional organ volume receiving a dose D_*i*_ with the equivalent dose given in 2 Gy fractions. Separately, a sensitivity analysis was performed to determine the impact of small changes to the treatment design.

The normalized total dose was used to estimate the impact on the GTV and PTV, based on the following equation:

$$ N T D = n{\displaystyle {\sum}_i}{v}_i{d}_i\ \left(\frac{1 + \frac{d_i}{\alpha /\beta}}{1 + \frac{2}{\alpha /\beta}}\right) $$

where, n, v_*i*_, and d_*i*_ represent the number of fractions (i.e. 10), the fractional volume *i* receiving a discretized dose at I, respectively. The alpha-beta ratio (α/β) used in the NTD calculation was 10, while an α/β of 3 was used for all normal tissue calculations. Dose volume histograms were exported and read using Matlab (R2012b, Mathworks, MA) where dosimetric parameters such as the mean dose to the esophagus and lung were determined. Comparisons were made using the Wilcoxon matched pair signed rank test. All statistical analyses were performed using SAS software (versions 9.3, SAS institute, CARY, USA), using two-sided statistical testing at the 0.05 significance level.

## Results

A total of 15 patients met the inclusion criteria. The mean GTV and PTV volumes were 97.6 cc [22.3–199.8] and 334.8 cc [164.1–564.9], respectively. Most cases (*n* = 12) had a GTV directly abutting the esophagus; all but one patient had a compromised PTV. The average field size of the POP plan was 178 ± 67 cm^2^, while the average length of the esophagus contained within the field (D50) was 12 ± 3 cm.

**Fig. 1:**
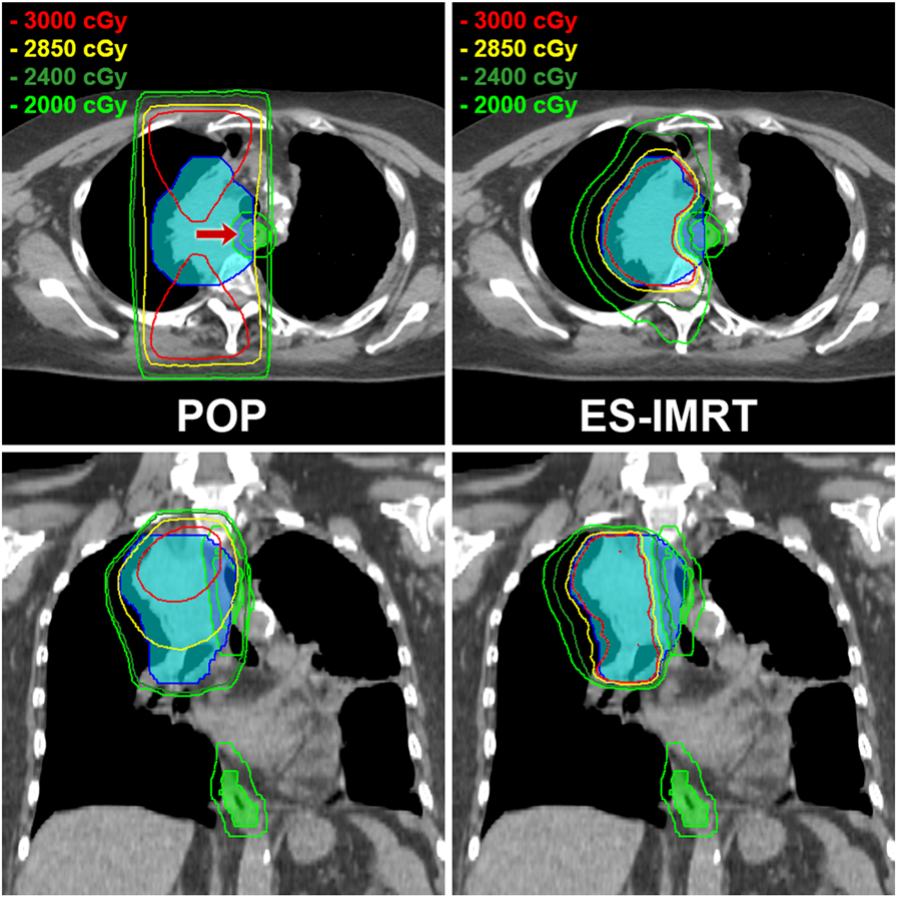
Axial and coronal CT slices illustrating the isodose distributions of the competing POP and ES-IMRT plans. The *blue* contour shows the PTV and overlap region (indicated by a *red* arrow) of the esophagus and esophagus ring having a *green* overlay and *green* contour, respectively. The *turquoise* colour overlay represents the uncompromised PTV (the ESPTV)

**Fig. 2:**
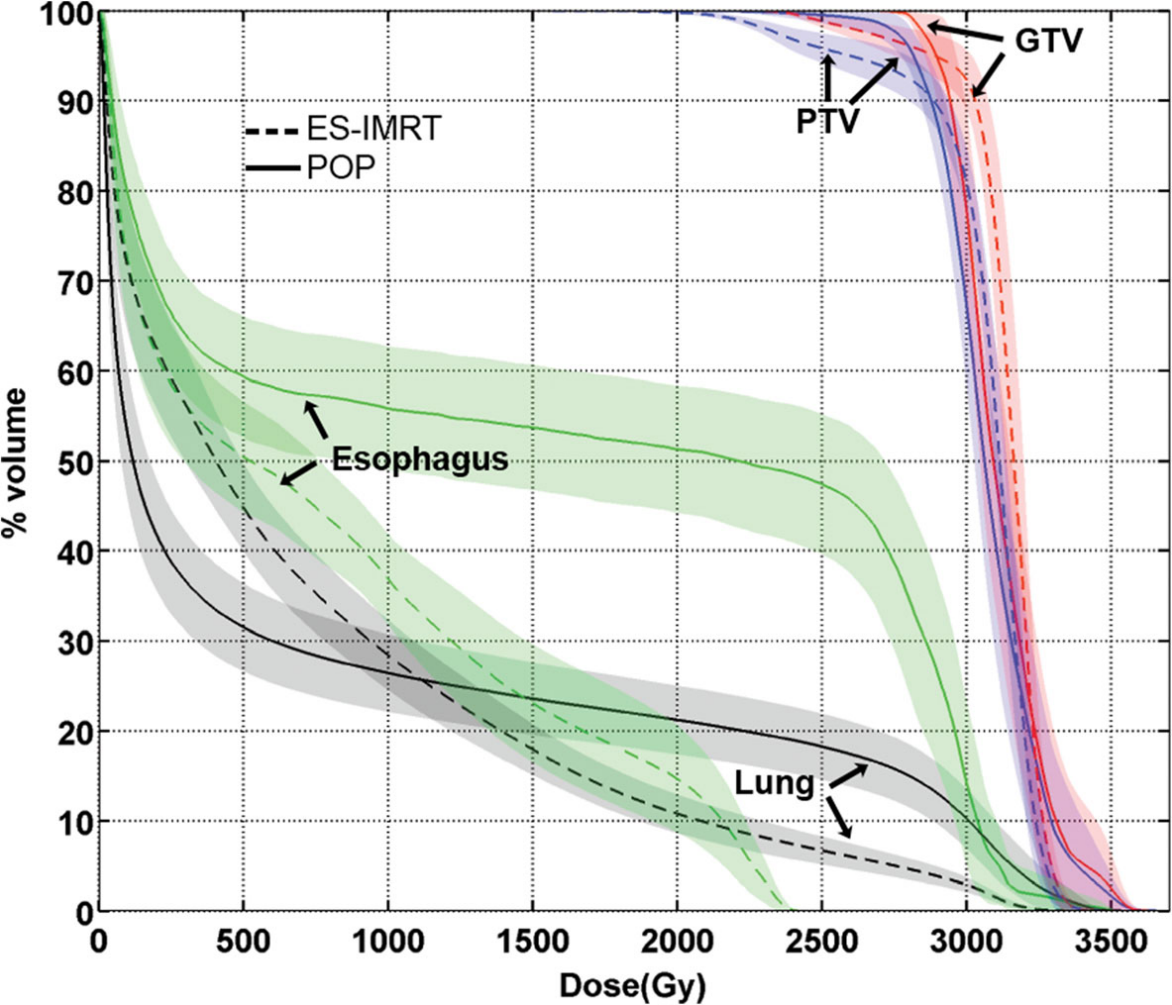
Average cumulative dose-volume line plots of the GTV (*red*), PTV (*blue*), esophagus (*green*), and lung (*black*) are shown for the clinically delivered POP plans (*solid line*) compared to the ES-IMRT optimized plans (*dashed line*) for the 15 patients meeting the inclusion criteria. The shaded envelope surrounding each line plot represents one standard deviation

Using ES-IMRT, the cohort mean esophageal dose was reduced from 16 Gy to 8 Gy (*p*-value < 0.001). Reductions in cohort mean lung dose from 8 Gy to 7 Gy were not statistically significant (*p* = 0.229). A representative example of the two competing plans in one patient can be seen in Fig. 1. The average and standard deviation of the cumulative dose-volume plots for the GTV, PTV, esophagus, and healthy lung for all the completing plans are shown in Fig. 2. The difference in the average GTV D95 between treatment arms was within 1%; however, the difference of the average PTV D95 was 8% lower in the ES-IMRT arm. Dose-volume metrics based on the plots in Fig. 2 are tabulated in Table 1.

**Table 1:** Dosimetric parameters describing the differences between the standard parallel-opposed pair beam arrangement vs. the proposed esophageal-sparing ES-IMRT plans

Parameter	Parallel-opposed pair plan	ES-IMRT esophagus sparing plan
D99 (GTV)	28.0 ± 1.0 Gy	24.7 ± 1.4 Gy
D95 (GTV)	28.9 ± 8.8 Gy	28.8 ± 4.8 Gy
D5 (GTV)	34.0 ± 14 Gy	32.9 ± 7.4 Gy
D99 (PTV)	26.5 ± 2.5 Gy	22.0 ± 0.8 Gy
D95 (PTV)	28.1 ± 4.6 Gy	25.9 ± 3.6 Gy
D5 (PTV)	33.8 ± 12 Gy	32.7 ± 4.4 Gy

Based on published LBK-NTCP models and the 15 patients studied herein, the mean probability of symptomatic esophagitis and pneumonitis reduced from 13 to 2%, and from 5 to 3%, respectively, when using ES-IMRT. Differences in mean predicted rates of toxicity for the competing plans were found to be statistically significant for both esophagitis (*p*-value < 0.001) and pneumonitis (*p*-value = 0.005). Earlier LKB-models of esophagitis by Belderbos et al. and Chapet et al. produced toxicity rates of 7% and 6% for the POP arm, which equally reduced to 1% in the ES-IMRT, suggesting that although the absolute rates of toxicity may vary using fit parameters derived from different insitutions, the relative reduction in the rates of observed toxicity are similar when invoking an esophagus-sparing strategy and LKB models.

The mean NTD for the GTV and PTV of the clinically approved POP plan versus the ES-IMRT plans were 34.0 ± 1.5 Gy and 33.7 ± 1.3 Gy, versus 34.3 ± 0.9 Gy and 33.3 ± 0.8 Gy, and were not found to be found statistically different (*p*-value = 0.277) and (*p*-value = 0.847), respectively.

## Discussion

Advanced radiotherapy techniques such as ES-IMRT may have clinical utility in reducing treatment-related toxicity in patients with advanced lung cancer. This study suggests that when considering a dose of 30 Gy in 10 fractions, the rate of esophagitis may be reduced from 13 to 2%, while maintaining a similar normalized total dose to the PTV. The lower calculated toxicity is a consequence of reducing the dose to the esophagus through the use of a ring structure that limited the maximum dose near the esophagus, as well as a minimum dose when the PTV overlapped with the ring structure, which occurred for all but one of the patients. Although, the earlier LKB-NTCP models by Belderbos et al. and Chapet et al. resulted in lower absolute toxicities using parallel opposed fields the relative reduction using ES-IMRT for all three evaluted NTCP models was similar between 5 and 7 times lower. The impact of the minimum dose to the PTV of 20 Gy on the treatment planning raised the likelihood of esophagitis from 1 to 2% as reported. This incremental level of the probability of esophagitis was felt to be a reasonable tolerance in order to maintain coverage.

A partial dose reduction to the overlap region of the esophagus and PTV in curative-intent patients is controversial but has been recently proposed; and, based on these reports, biological models suggest a meaningful reduction in the likelihood of esophagitis without a compromise in tumor control [9, 10].

Considering that the clinical objective for the patients under investigation in this study is symptom relief over local control, we believe that this cohort of patients represents an interesting group from which to evaluate the impact of partial PTV and GTV compromises. In particular, patients treated with a compromised PTV may reveal meaningful changes in quality of life that would not have otherwise been recorded as a grade II toxicity requiring medical intervention. Quite promisingly, in a contralateral esophageal-sparing technique, 20 curative-intent patients experienced no grade 3 adverse, 4 patients recorded a grade 2 adverse event, and 11 patients recorded a grade 1 adverse event [3]. Given the limited sample size and follow-up in this cohort, further research is required to assess whether such a strategy is generalizable and/or feasible in the broader patient population.

Dysphagia induced as a result of esophagitis can cause significant discomfort to the patient, and even place additional stress on caregivers to ensure adequate nutrition is being administered. Dysphagia has a multifactorial etiology and differences exist in its assessment rating between patient reported difficulty versus clinician ascribed [11]. Regardless, for curative-intent patients dysphagia has been associated with inferior outcomes; therefore, strategies to help mitigate radiation-induced associate esophagitis through IMRT may improve the efficacy of treatment and overall quality of life.

## Conclusion

In conclusion, ES-IMRT may be useful in reducing dysphagia, but clinical data are needed to confirm the model-predicted benefits reported herein. Our data have formed the basis for the design of a randomized phase III study of Palliative Radiation of Advanced Central lung Tumors with Intentional avoidance of the Esophagus (PROACTIVE), which is actively accruing patients with the goal of assessing the clinical benefit of ES-IMRT.

## Abbreviations

CTV: Clinical tumor volume
ES-IMRT: Esophageal sparing intensity modified radiation therapy
GTV: Gross tumor volume
ITV: Internal target volume
LKB-NTCP: Lyman-Kutcher-Burman normal tissue complication probabilities
NSCLC: Non small cell lung cancer
NTD: Normalized total dose
OAR: Organs at risk
POP: Parallel-opposed pair
PTV: Planning target volume
RCT: Randomized controlled trials
RTOG: Radiotherapy oncology group
VMAT: Volumetric modulated arc therapy
